# Protocol for regional implementation of community-based collaborative management of complex chronic patients

**DOI:** 10.1038/s41533-017-0043-9

**Published:** 2017-07-14

**Authors:** Isaac Cano, Ivan Dueñas-Espín, Carme Hernandez, Jordi de Batlle, Jaume Benavent, Juan Carlos Contel, Erik Baltaxe, Joan Escarrabill, Juan Manuel Fernández, Judith Garcia-Aymerich, Miquel Àngel Mas, Felip Miralles, Montserrat Moharra, Jordi Piera, Tomas Salas, Sebastià Santaeugènia, Nestor Soler, Gerard Torres, Eloisa Vargiu, Emili Vela, Josep Roca

**Affiliations:** 1 Hospital Clinic de Barcelona, Institut d’Investigacions Biomèdiques August Pi i Sunyer (IDIBAPS), Universitat de Barcelona, Barcelona, Spain; 2 Center for Biomedical Network Research in Respiratory Diseases (CIBERES), Majadahonda (Madrid), Spain; 3 ISGlobal, Centre for Research in Environmental Epidemiology (CREAL), Universitat Pompeu Fabra (UPF), CIBER Epidemiología y Salud Pública (CIBERESP), Barcelona, Spain; 4 Respiratory Department, Institut de Recerca Biomedica (IRBLeida), Lleida, Spain; 5 Consorci d’Atenció Primària de Salut Barcelona Esquerra (CAPSBE), Barcelona, Spain; 6 Departament de Salut, Generalitat de Catalunya, Barcelona, Catalonia Spain; 7 Eurecat. Technological Center of Catalonia, Barcelona, Catalunya Spain; 8 Badalona Serveis Assistencials (BSA), Badalona, Catalonia Spain; 9 Agència de Qualitat i Avaluació Sanitàries de Catalunya (AQuAS), Barcelona, Catalonia Spain; 10 CatSalut, Servei Català de la Salut, Barcelona, Catalonia Spain

## Background

Over the last few years, the epidemics of noncommunicable diseases and the need for cost-containment^1^ are triggering factors for a profound transformation of the way we approach delivery of care for chronic patients. In this new scenario, conventional disease-oriented approaches, centered on the management of clinical episodes, are being replaced by patient-centered integrated care services,^2^ as promoted by the World Health Organization.^3^

Lessons learnt from deployment experiences^4, 5^ following patient-centered approaches are being disseminated as good practices.^6^ However, there are several factors that need further attention, such as the need for further assessment of implementation strategies in real-world scenarios and the lack of transferability from progress achieved in disease-oriented integrated care to management of complex chronic patients (CCP).^5, 7^ Likewise, efficacy achieved in integrated care interventions, assessed through randomized controlled trials,^8–11^ may not translate into effectiveness at health system level.^12^

In addition, poor comparability among experiences on management of multimorbidity emerges as an important hurdle for the adoption of integrated care. In this regard, the lack of an operational definition for CCP is not a negligible factor, as it clearly limits an appropriate service workflow design, which, in turn, precludes both evaluation and comparability of reported experiences.^5–7^

The term CCP is usually applied to subjects with heterogeneous conditions that may depict at least one of the following three traits: (i) need for management by several specialists from different disciplines generating high use of healthcare resources; (ii) frailty,^13^ requiring additional support either due to functional decline, social deficits, and/or transient situations like post-hospital discharge^14–16^; or, (iii) need for highly specialized care with home-based technological support.^4^ Moreover, CCP often show a dynamic evolution over time in terms of both health risk and care requirements,^6^ such that their management requires a balance between planned (predefined and repeatable) and unplanned processes (depending on evolving circumstances and ad-hoc decisions).

The CCP protocol relies on the hypothesis that implementation of (i) structured, but flexible service workflows, that is, a collaborative and adaptive case management approach^17^ and (ii) enhanced patient health risk assessment and stratification can overcome current limitations of multimorbidity management. The protocol for management of CCP aims to assess the study hypothesis through the evaluation of the regional deployment of two existing integrated care interventions (i.e., implementation studies) described below. It is of note that the protocol evaluation will also include a population-based analysis of CCP management.

The two implementation studies have a quasi-experimental design. That is, a nonrandomized intervention group (integrated care) is compared to a control group (usual care) using propensity score methods^18^ wherein age, gender, and population-based health risk assessment are the main variables to be used for adjustment. The Catalan population-based health risk assessment tool (GMA, Adjusted Morbidity Groups)^19, 20^ will be used for health risk scoring purposes. The protocol evaluation follows a Triple Aim approach^21, 22^ considering predefined outcome variables for (i) health and well-being, (ii) experience with care, and (iii) costs. Assessment will be carried out combining empirical questionnaire data collection, information from electronic medical records, and registry data. The main study outcome will be twofold: (i) demonstration of cost-effectiveness of the interventions; and, (ii) identification of factors that modulate success of large-scale deployment. A post-hoc statistical power analyses for assessment of the main protocol outcomes will be done.

## Aims

**Fig. 1:**
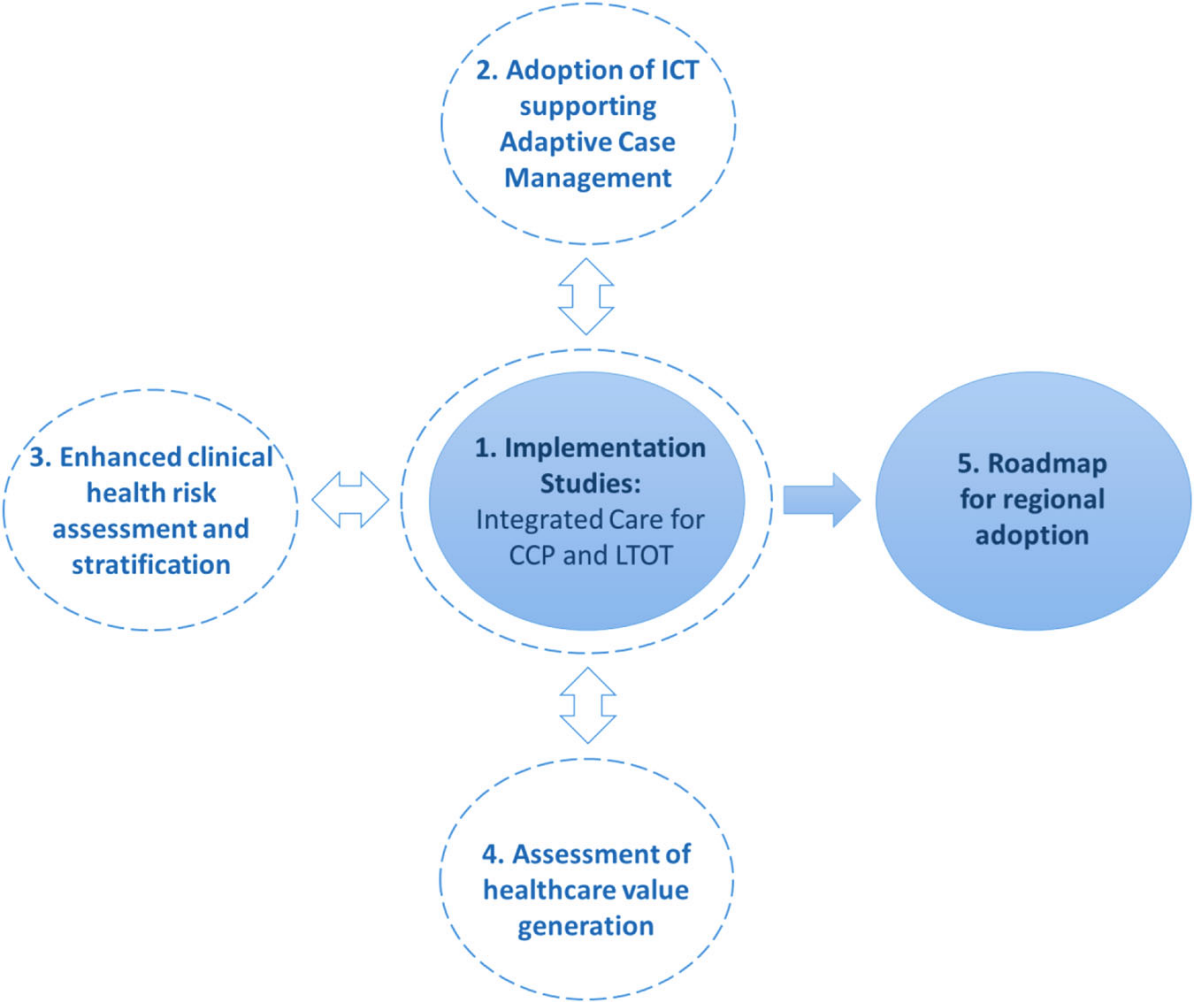
Aims of the study. Five pivotal aims to achieve successful regional adoption of the community-based protocol for collaborative management of complex chronic patients across healthcare tiers

The protocol addresses the five aims displayed in Fig. 1. Firstly, execution of implementation studies of two integrated care interventions with proven efficacy in previous studies^4^: (i) Community-based management of CCP and, (ii) Integrated care for patients under long-term oxygen therapy (LTOT). The two implementation studies will allow (second aim) to assess the impact of collaborative adaptive case managment^17^ supported by information and communication technologies. The third aim of the protocol is to evaluate the impact of enhanced clinical health risk assessment and stratification^19^ on the two implementation studies. The fourth aim is assessment of healthcare value generation^23, 24^ of the interventions, both during the deployment phase and after regional scaleup of the services. Finally, the current study will generate a roadmap for regional adoption of the CCP protocol.

## Discussion

The current protocol describes a comprehensive strategy for achieving regional adoption of integrated care for complex chronic patients in Catalonia. It relies on two main innovative pillars: (i) Technological platforms to support service workflows based on collaborative and adaptive case management of CCP; and, (ii) Highly transferable enhanced clinical risk assessment and stratification strategy. To our knowledge, the current protocol design shows key factors required to overcome limitations observed in other regional deployments.

In summary, the CCP protocol articulates lessons learnt in previous experiences^4^ carried out and validated in Catalonia during the last few years in order to generate a collaborative ecosystem with high potential for transferability to other geographical areas. The main project outcome will be generation of guidelines for large-scale deployment of the CCP protocol, including transferability analysis that shall facilitate adoption of cost-effective integrated care services for management of multimorbidity.

## Methods

### The setting

The current manuscript describes the protocol for large-scale deployment of integrated care services for CCP undertaken in the healthcare sector of Barcelona-Esquerra (520k citizens) and in two other areas of Catalonia: Badalona Serveis Assistencials (420k citizens) and Lleida (366k citizens). The protocol for regional deployment of CCP management in Catalonia (ES) (7.5M citizens) is being developed under the umbrella of the Catalan Government Health Plan 2016–2020,^25^ and it is supported by the convergence of resources among innovation plans of healthcare providers, public resources, and manpower from different grants.^26^ The research was submitted to the Ethical Committee of the Hospital Clínic of Barcelona, and it has been registered as at clinicaltrials.gov (NCT02956395—Implementation of Community-based Collaborative Management of Complex Chronic Patients (Nextcare_CCP)).

**Fig. 2:**
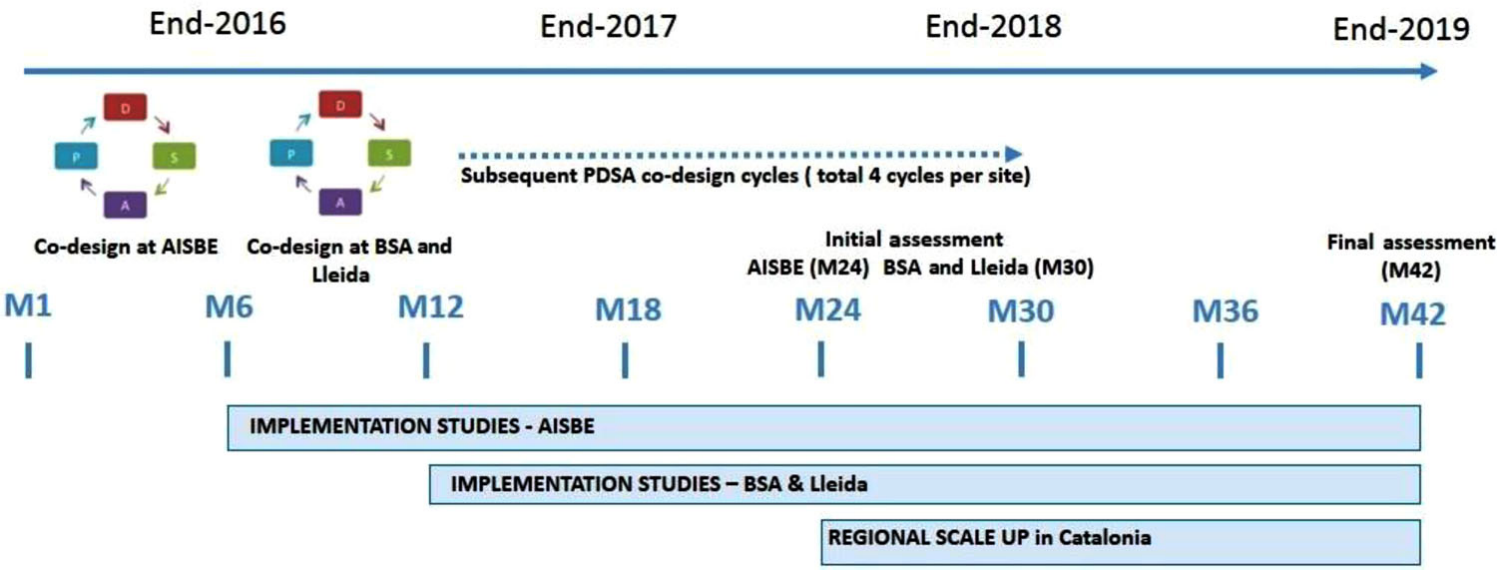
Timeline of the protocol for large-scale deployment of CCP management in Catalonia. AISBE stands for integrated care area of Barcelona-Esquerra and BSA is the abbreviation of Barcelona Serveis Assistencials. A total of four codesign cycles, of 6-month duration each, are planned in each site. At the end of the assessment of the implementation studies (M42), a consensus on key performance indicators for follow up of integrated care interventions’ adoption beyond the current study will be achieved

In the other two sites (Lleida and Badalona), the two implementation studies will begin by mid-2017 in order to facilitate site adaptation of the service workflows. An initial assessment of all implementation studies will be done after 18 months of the trials initiation in each of the sites. The timeline of the CCP integrated care intervention is indicated in Fig. 2.

### Implementation studies

While the total protocol duration will be 42 months, from mid-2016 to end of 2019 (Fig. 2), on January 2017, two implementation studies were initiated at Barcelona-Esquerra: (i) Community-based management of CCP (intervention group, *n* = 3000) and (ii) Integrated care for patients under LTOT (intervention group, *n* = 500).^27^ A codesign process (Supplementary Methods) following a Plan–Do–Study–Act methodology^28^ will be carried out in each site by a multidisciplinary team including: primary care professionals (general practitioners, nurses, and social workers), specialists (doctors and allied health professionals), technologists, patients, and caregivers.

#### The integrated care intervention for community-based management of CCP

**Fig. 3:**
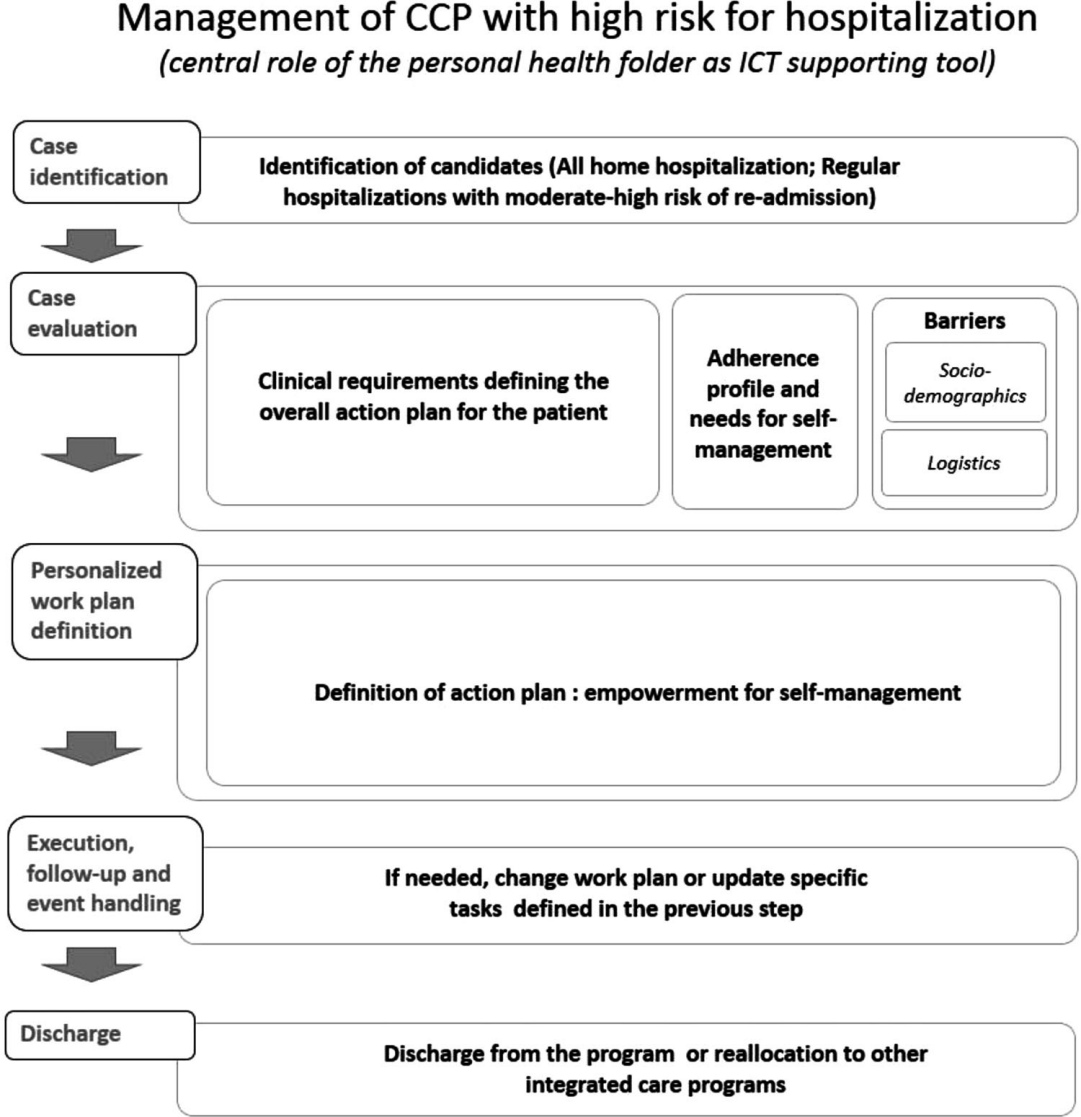
General service workflow. General description of the service workflow through six sequential steps

The integrated care intervention for community-based management of CCP constitutes the core implementation study in the protocol. The service workflow (Fig. 3) has two sequential phases with specific target outcomes for each of them: (i) Short-term intervention to prevent early (30 and 90 days) hospital-related events; and (ii) Intervention to enhance community-based long-term management of CCP.

Eligible candidates for inclusion are patients showing moderate-to-high risk of early readmission (LACE index ≥ 7)^29^ recruited immediately after hospital discharge. Additional inclusion criteria to be fulfilled by candidates are as follows: (i) living in his/her house within the healthcare sector; (ii) having a phone at home; and, (iii) signing a written acceptance form to participate in the implementation study. The exclusion criteria for the study are patients with severe psychiatric or neurologic disorders impeding patient collaboration.

The intervention is implemented by a multidisciplinary team from the hospital and primary care consisting of advanced practice nurses, physicians, physiotherapists, community nurses, and social workers, having a general practitioner as a reference. The collaboration between specialized care and primary care is guided by the reform of specialized care in the healthcare sectors initiated in 2006. This reform^30^ aimed to improve healthcare quality and accessibility, based on coordination between the Hospital Clínic and the different suppliers to the integrated health area of Barcelona-Esquerra. The challenge for Hospital Clinic is to work simultaneously as a dual hospital: high-tech (as a national reference center) and as a community hospital for the healthcare sector. For the development of the reform, a follow-up body was set up with differentiated working groups to define the organizational structure, the information systems requirements, and the care processes.

The intervention during hospital admission includes a comprehensive assessment of the patient at entry including severity of the primary disease, evaluation of comorbid conditions and analysis of social support needs. Moreover, a 2-h educational program is administered by a nurse followed by distribution of patient-specific support material. The educational program covers knowledge of primary disease and comorbidities, instructions on nonpharmacological treatment, administration techniques for proper pharmacological therapy, and techniques for self-management of the disease along with comorbid conditions, including strategies to prevent future severe exacerbations.

The intervention includes a phone call at 24 h and a home visit at 72 h after hospital discharge by one member of the multidisciplinary team, if needed. During this home visit, the therapeutic plan for each patient will be customized to their individual frailty factors and shared with the primary care team. Reinforcement of the logistics for treatment of comorbidities and social support will be done accordingly. Moreover, the personal health folder will be used for patient empowerment for self-management and as a tool to facilitate accessibility to health professionals. The personal health folder of Catalonia (https://lamevasalut.gencat.cat) provides citizens with an access point to their clinical information (i.e., electronic prescriptions, administered vaccines, diagnosis, clinical reports, and diagnostic/lab tests) and can also act as the citizen entry point for some of the supported administrative processes (e.g., appointments), interactions with health professionals, and potentially for connection with informal health data sources (e.g., mobile health applications and community social support services).

The advance-practice nurses perform regular training sessions to the community-based care teams, coordinate accessibility to specialized care as needed, and support functionalities of the personal health folder for the patients admitted into the protocol. The number of home care visits, as well as access to specialized care during the follow-up of a 12-month period, is individually tailored, and dynamically adapted, to patient needs. Moreover, planned visits by specialized professionals can be scheduled through day hospital or home visits if primary care teams consider this necessary.

#### The integrated care intervention for management of patients currently under LTOT

**Fig. 4:**
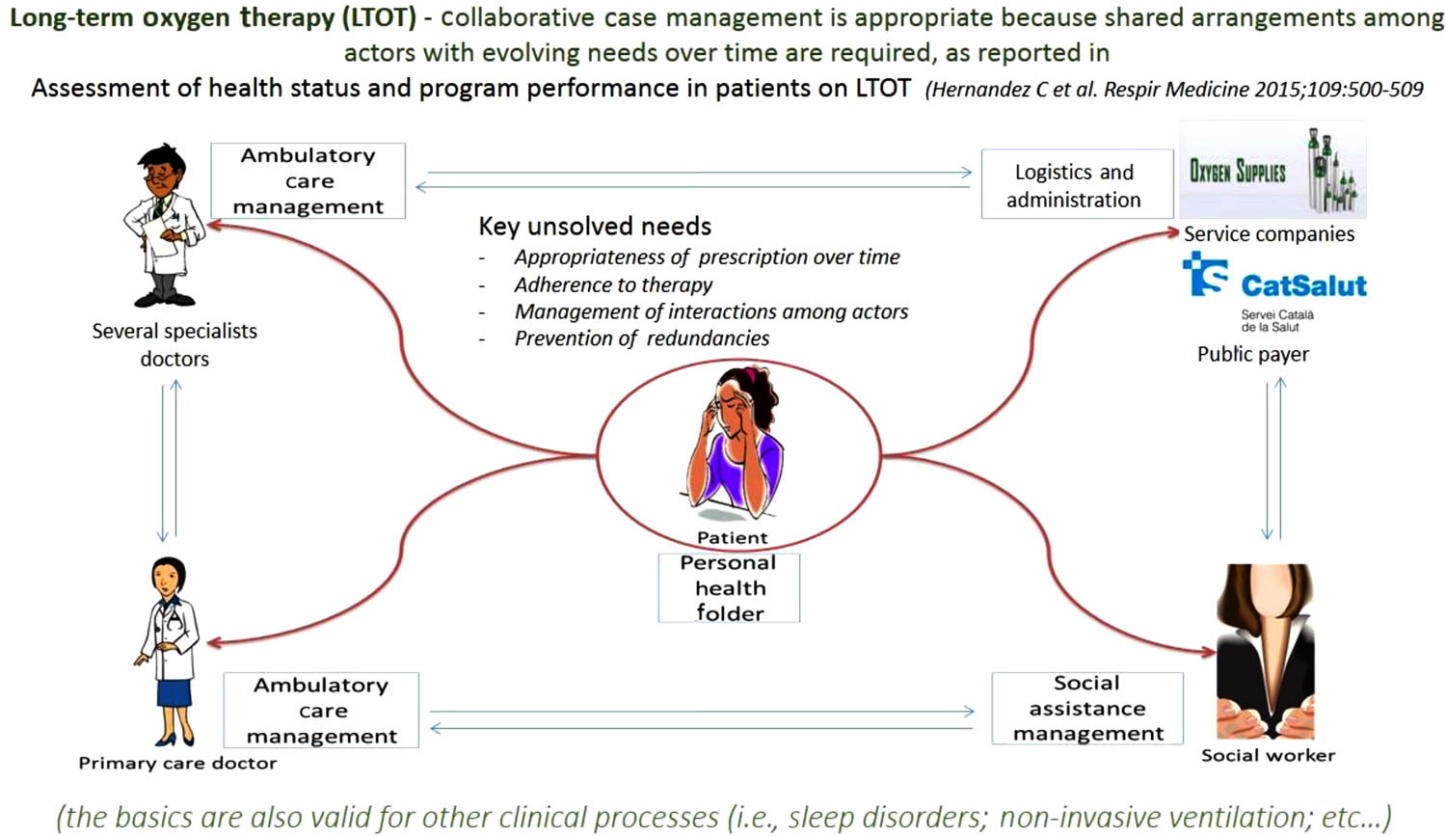
Integrated Care Management of patients under LTOT

The integrated care intervention for management of patients currently under LTOT was selected for inclusion in the current protocol because patients under LTOT constitute a representative group of frail multimorbid individuals requiring cooperative management of multiple actors including community-based health care professionals, specialists, and companies providing home-based services (Fig. 4). The characteristics and unmet needs of the LTOT group of patients in Barcelona-Esquerra have been described in detail elsewhere.^27^ The focus of the study is the analysis of the impact of technological tools supporting collaborative management on main outcomes, namely: (i) adequacy of prescription; (ii) adherence; and, (iii) enhanced community-based management of the patients.

### Adoption of ICT supported adaptive case management

The general structure of the service workflows in the protocol encompasses five main steps: (1) Case identification—It refers to identification of candidates for inclusion into the protocol following predefined inclusion/exclusion criteria; (2) Case evaluation—Initial holistic characterization of the patient, including health risk assessment, done at entry into the program; (3) Personalized work plan definition—Elaboration of a personalized action plan based on case evaluation; (4) Work plan execution, follow-up and event handling—Execution of the work plan will be done with technological support to facilitate the protocol follow-up and the handling of unexpected events by (i) fostering patient empowerment for self-management; (ii) enhancing patient adherence to the protocol; (iii) facilitating remote supervision; (iv) allowing patient monitoring; and (5) Discharge—At the end of the protocol evaluation (Fig. 2), the patient can remain in the program or he/she can be moved to other types of integrated care services depending upon his/her needs.

Adoption of adaptive case management^17, 31^ to support collaborative work constitutes an emergent approach that facilitates case managers to adapt well-structured service workflows to the continuously evolving needs of the patients. This implies selection and scheduling of specific tasks during case management and ad-hoc collaboration with other professionals across healthcare and social support tiers, which facilitates collaborative decisions triggered by expected and unexpected events.

Therefore, the two target-integrated care interventions will be supported by a software platform that will allow the execution of well-structured but adaptable clinical workflows. This platform will be open-source and built-up on top of the current health information systems of the different healthcare providers and using existing regional interoperability infrastructures. In order to support both patient collaborative work and self-management, the personal health folder already deployed in the region is currently being adapted for the purposes of the protocol as a key component of the Catalan Digital Health Framework.^32^

### Enhanced health risk assessment and stratification

**Fig. 5:**
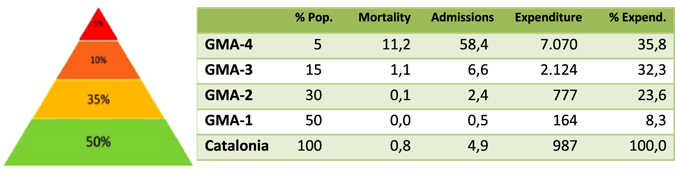
Stratification of the Catalan population (2014) using the GMA. Previously published in Dueñas-Espín I et al., BMJ Open. 2016 Apr 15;6(4) as part of the online supplementary material (Supplementary Figure 2S). The third and fourth columns depict rates of mortality and hospital admissions, respectively. The fifth column indicates the cost per inhabitant per year expressed in € and the last column refers the percentage of total healthcare expenditure by risk strata. It is of note that the closer the patient is to the tip of the pyramid, the higher are mortality, risk of hospital admission, and healthcare expenses. *Green color* (*bottom*) indicates healthy status, whereas *red* (*tip*) corresponds to maximum risk of admissions and highest mortality risk

The CCP protocol acknowledges that health risk prediction and stratification is a relevant driver for large-scale deployment of integrated care.^6^ Accordingly, the project will use the regional population health risk assessment tool (GMA) to enhance clinical risk assessment and stratification (Fig. 5).

Prospective assessment of both practicalities and added value of the use of GMA scoring for enhanced clinical risk prediction and stratification, as reported in detail elsewhere,^19^ has been successfully completed in Catalonia for the entire population of patients with Chronic Obstructive Pulmonary Disease (unpublished). This approach will be incorporated in the implementation studies undertaken into the current CCP protocol aiming at enhancing risk prediction in the clinical scenario.

The GMA tool predicts individual patient risk based on multimorbidity information gathered from the Catalan Health Surveillance System described below. The rationale behind the use of GMA, against alternative health risk assessment tools, is that it complies with four main recommended criteria^19^; that is, (i) a population health approach (using the entire source population of 7.5 million inhabitants of the region); (ii) publicly owned without licensing constraints; (iii) open-source computational algorithms; and, (iv) the GMA morbidity grouper relies on statistical criteria, as opposed to other tools that include expert-based coefficients, thus facilitating quick transferability to other territories, as recently shown in Spain wherein GMA risk assessment is used in 38 million inhabitants.

### Assessment of healthcare value generation

The evaluation strategy (see Supplementary Methods for further details) has a threefold aim: (i) identification of factors that modulate large-scale deployment of the two implementation studies in the three sites; (ii) assessment of cost-effectiveness of the intervention for enhanced community-based management of CCP; and (iii) evaluation of the added value of the technological support for integrated care management of patients under LTOT. To this end, we will use implementation research tools^33, 34^ organized within the frame of the Model for ASsesment of Telemedicine applications.^35^ As alluded to above, the two implementation studies have a quasiexperimental design wherein integrated care (intervention) will be compared to usual care (control) with age, gender, and GMA scoring as main matching variables. Moreover, a population-based analysis of CCP management using registry data from the Catalan Health Surveillance System (CHSS) (Supplementary Fig. 1) will be carried out assessing separately intervention and control areas in each of the three sites.

Predefined outcome variables (Table 1) will include the eleven indicators recommended by the Spanish Health System^36^ for assessment of four care coordination categories of variables. At the end of the study, we will propose Key Performance Indicators, selected among those used in the regional deployment phase, to be considered for population-based assessment of integrated care interventions for CCP beyond the lifespan of the study.

**Table 1:** Predefined outcome variables for evaluation purposes selected with a “Triple Aim” approach

Triple Aim	Outcome	Data source and Instrument
Health and well-being	Sociodemographics	Catalan Health Surveillance System and Electronic Medical Records
	Multimorbidities	Catalan Health Surveillance System and Electronic Medical Records
	Patient Clinical Data	Electronic Medical Records
	Health-related quality of life	SF-36 questionnaire^37^
	Therapeutic plan (Pharmacological/Others)	Catalan Health Surveillance System and Electronic Medical Records
	Intermediate outcomes (see costs):	Catalan Health Surveillance System
	• Emergency department visits	
	• General practitioner visits	
	• Cumulative days per year admitted in hospital	
	• Multiple drugs’ prescription	
	• Potentially avoidable hospitalizations	
	• Hospital readmissions	
	• Needs for social support	
	Mortality	Catalan Health Surveillance System/Electronic Medical Records
	Physical functioning	Short Form 36 (SF-36)^37^ ^, (1)^ or Katz-15^38^ ^, (1)^
	Psychological well-being	Mental Health Inventory (MHI-5) of the Short Form 36^37^ ^, (1)^
	Social relationships and participation	Impact on Participation and Autonomy (IPA)^39^ ^, (1)^
	Enjoyment of life	Investigating Choice Experiments for the Preferences of Older People (ICECAP-O)^40^ ^, (1)^
	Resilience	Brief Resilience Scale (BRS)^41^ ^(1)^
	Autonomy	Pearlin Mastery Scale^42^ ^, (1)^
	Activation and engagement	Short form Patient Activation Measure (PAM-13)^43^ ^, (1)^
**Experience with care**	Person centeredness	Person-Centered Coordinated Care Experiences Questionnaire (P3CEQ)^44^ ^, (1)^
	Continuity of care	Nijmegen Continuity Questionnaire (NCQ)^45^ ^(1)^
	Burden of medication	Living with Medicines Questionnaire (LMQ)^46^ ^, (1)^
	Burden of informal caregiving	Informal Care Questionnaire^47^ ^, (1)^
	Use of the Personal Health Folder	Catalan Health Surveillance System
	Access to integrated care	Catalan Health Surveillance System
	Healthy lifestyle (Tobacco/Nutrition/Alcohol/Physical Activity)	Electronic Medical Records
	Knowledge of current morbid conditions	Electronic Medical Records (nonstandard questionnaire)
	Multiple drug therapy	Catalan Health Surveillance System and Morinsky-Green questionnaire^48^
	Home-based technological support	Electronic Medical Records
	Patient satisfaction and engagement	Electronic Medical Records (nonstandard questionnaire)
	Caregiver satisfaction and engagement	Electronic Medical Records (nonstandard questionnaire)
**Costs** ^**(2)**^	Total health and social care cost	Catalan Health Surveillance System
	Primary Care	Catalan Health Surveillance System
	Hospital-related Care	Catalan Health Surveillance System
	• Admissions	
	• Emergency room consultations	
	• Outpatient specialized care	
	Pharmacy	Catalan Health Surveillance System
	Mental Health	Catalan Health Surveillance System
	Sociosanitary services	Catalan Health Surveillance System
	Other costs	Catalan Health Surveillance System
	• Respiratory therapies	
	• Dialysis	
	• Rehabilitation	
	• Nonurgent patient transport	

^(1)^ Questionnaire administered within the EU project SELFIE (see methods in Supplementary Section 1 for further details)

^(2)^ The Catalan Health Surveillance System registries (Supplementary Figure 1) allow allocation of healthcare expenditure to each patient through the Personal Health Identification Number, which facilitates analysis of total healthcare expenditure in complex patients

Data for evaluation purposes, for both implementation studies and population-based analysis (Table 1 and Supplementary Fig. 1), will be obtained from (i) Electronic Medical Records; (ii) the CHSS; and, (iii) Standardized questionnaires selected with a “Triple Aim” approach,^21, 22^ this is, considering predefined outcome variables for (i) health and well-being, (ii) experience with care, and (iii) costs.

The CHSS (Supplementary Fig. 1) includes updated registries from Primary Care, Hospital-related events (hospitalization, emergency room, and specialized outpatient visits), Pharmacy, Mental Health, Sociosanitary services, respiratory therapies, dialysis, outpatient rehabilitation, nonurgent health transport, outpatient minor surgery, home hospitalization and implants since 2011. It allows analyses on use of healthcare resources, pharmacy consumption, and prevalence of key disorders. The CHSS also feeds the population-based health risk assessment tool known, GMA, that is used to elaborate the health risk strata pyramid of the general population of Catalonia,^19, 20^ which is periodically updated on a 6-month basis. The CHSS allows allocation of healthcare expenditure, including pharmacy, to each patient through the Personal Health Identification Number, which facilitates analysis of total healthcare expenditure in complex patients.

### Data availability

Data sharing is not applicable to this article as no data sets were generated or analyzed during the current study.

## Electronic supplementary material

Supplementary Methods

Supplemetary Figure 1
