# Supplementary material for: Protocol for regional implementation of community-based collaborative management of complex chronic patients
Source: NPJ Prim Care Respir Med. 2017 Jul 14;27:44. doi: 10.1038/s41533-017-0043-9 (PMC5511202; doi:10.1038/s41533-017-0043-9)
Supplement: Supplementary file 1 — Supplementary Methods [file 41533_2017_43_MOESM1_ESM.docx]

# Protocol for Regional Implementation of Community-based Collaborative Management of Complex Chronic Patients

*Isaac Cano et al*

*On-line supplementary material*

# Section 1 – Expanded description of CCP protocol evaluation

Poor comparability among interventions carried out in different studies constitutes one of the most important hurdles for assessing effectiveness and health value generation of integrated care for Chronic Complex Patients (CCP) management. The current section complements the assessment strategy described in the main manuscript, ***Protocol evaluation***, by providing detailed descriptions of outcome variables and measurement instruments (**Table 1** and **Supplementary** **Figure 1**) used for evaluation of the two implementation studies undertaken in the CCP protocol and for the population-based analysis. We also describe details of the study design, sub-studies and logistics in the three deployment sites. Moreover, the section addresses methodological aspects of the co-design process.

## Outcome variables and data sources

The selection of outcome variables agreed during the first co-design cycle (**Figure 2**) is based on a “Triple Aim” ^1,2^ approach. Thus, it covers the following dimensions (**Table 1**): (i) health and well-being, (ii) experience with care, and (iii) costs. It is of note that the list of proposed outcome variables also includes the eleven indicators jointly suggested in 2016 by the Agency for Health Quality and Assessment of Catalonia and the Spanish Government to assess integrated care for chronic patients^3^. These eleven indicators are grouped in four categories of variables: i) Characteristics of the study groups and health status (i.e., 1. Users attended in primary care and, 2. Health-related quality of life of patients and caregivers); ii) Intermediate outcomes (3. Emergency Department visits; 4. General Practitioner visits; 5. Cumulative days per year admitted in hospital; 6. Poli-medication; 7. Potentially avoidable hospitalizations; 8. Hospital readmissions; 9. Needs for social support); iii) Empowerment (10. Unhealthy life styles); and, iv) Structure indicators of integrated care (11. Access to integrated care services).

As indicated in the main manuscript, one of the protocol outcomes will be the proposal of a set of Key Performance Indicators, selected among those used in the regional deployment phase (**Table 1**), to be considered for population-based assessment of integrated care interventions for CCP at regional level beyond the lifespan of the current protocol. Moreover, through the program lifespan (**Figure 2**) progresses consolidated by the International Consortium for Health Outcomes Measurement^4^ will be incorporated.

Data for evaluation purposes (**Table 1**) will be obtained from: i) Electronic Medical Records; ii) Catalan Health Surveillance System (CHSS) (**Supplementary** **Figure 1**); and, ii) Standardized questionnaires.

## Expanded description of the implementation studies at site level

In all three sites, the CCP protocol will be conducted combining two core modalities of analysis. Firstly, the two implementation studies will be evaluated in each of the three sites with a quasi-experimental design using propensity score matching^5^, as reported in the main manuscript and further described for each of the three sites in the current section. Data sources for this analysis are indicated in **Table 1**, namely: (i) registry data retrieved from the Catalan Health Surveillance System (CHSS) (**Supplementary** **Figure 1**); (ii) Electronic Medical Records; and, (iii) Standardized questionnaires.

The second level of analysis will follow a population-based approach, for which data will be retrieved from the CHSS (**Supplementary** **Figure 1**). The population-based analysis will compare intervention and control areas in each of the three sites. It will also perform sub-analysis to assess effects at provider level and primary care unit level, as allowed by the characteristics of the CHSS.

Moreover, clustered randomized controlled trials nested within the intervention arm (integrated care) of the quasi-experimental studies could be planned, for a given period, to test specific questions. For example, performance and added value of the technological platform providing adaptive case management functionalities, etc.

### Implementation Study on Community-based management of CCP

*Barcelona-Esquerra site -* The implementation study on ***Community-based management of CCP*** (n=3,000) will be conducted as described in the main manuscript. But, it will mostly focus on patients included in the program addressing Home Hospitalization/Early Discharge and Transitional Care run at Hospital Clinic since 2006, as intervention group, which constitutes the proposal of Barcelona-Esquerra for regional deployment of community-based management of CCP. The control group will include patients following identical inclusion/exclusion criteria among those admitted in the Hospital Sagrat Cor, a different provider in the same healthcare sector without neither Home Hospitalization/Early Discharge nor Transitional Care programs in place. The two groups will be matched by age, sex and Adjusted Morbidity Groups (GMA) health risk scoring. It is of note that a number of pre-defined questionnaires, indicated in **Table 1** as pertaining to the EU project SELFIE^6^, will be administered to a subset of randomly selected patients (n=600) from both the intervention and control groups. The specific purpose of the SELFIE’s questionnaires is to feed a Multiple Criteria Decision Analysis approach supporting new reimbursement modalities for services addressing CCP management.

*Badalona Serveis Assistencials site -* The implementation study on ***Community-based management of CCP*** (n= 1,000) will focus on individuals included in the home-based integrated care program for frail patients run at Badalona from 2005. The control group (usual care) will include patients following identical inclusion/exclusion criteria among those treated by the other main provider (Catalan Health Institute) in the same healthcare sector. The two groups will be matched by age, gender and GMA scoring. Also a number of pre-defined questionnaires, indicated in **Table 1**  as pertaining to the EU project SELFIE^6^, will be administered to a subset of randomly selected patients (n=500) from both the intervention and control groups.

*Lleida site -* The implementation study on ***Community-based management of CCP*** (n=200) will focus on individuals included in the integrated care program for frail chronic patients with multi-morbidity jointly conducted by Hospital Arnau de Vilanova and its primary care sector. In Lleida, the CCP protocol will have a pragmatic cluster randomized trial design with primary care units pertaining to the intervention group (integrated care) and others to the control group (usual care). The questionnaires indicated in **Table 1** as pertaining to the EU project SELFIE^6^ will not be administered in Lleida.

### Implementation study on integrated care for patients under long-term Oxygen therapy

Since integrate care is highly implemented in the three sites, all of them will be considered as part of the intervention group for the implementation study on ***integrated care for patients under long-term Oxygen therapy***, but each site will be analyzed separately. The corresponding control groups will be selected among areas with weak or no deployment of integrated care. The analysis will be carried out using registry data for respiratory therapies carried out by the Agency for Health Quality and Assessement of Catalonia.

## Co-design cycles

The Plan-Do-Study-Act (PDSA)^7^ co-design cycles will be conducted by a multidisciplinary group of stakeholders as described in the legend of **Figure 2**. The purposes of the co-design cycles are: i) Adjust the specifics of the service workflows to the characteristics of the healthcare sector before initiation of the implementation studies; ii) Refine the different dimensions assessed throughout the lifetime of the implementation studies such that a final version of the service workflows can be achieved after the initial 18-months of deployment; iii) assess suitability and acceptance of key performance indicators (KPIs) to assess the corresponding implementation study; and, iv) generate consensus on the KPIs to be used for long-term follow-up of the integrated care services beyond the project lifetime.

Plan-Do-Study-Act co-design cycles assessment instruments – Before the initiation of PDSA cycles working groups have been created to facilitate the success of the PDSA strategy. In this sense, a working team has been defined at each site for each of the implementation studies to be carried on in the site. Working teams are conformed of a variable number of highly motivated and fully engaged members, necessarily including the following:

- One local expert on the given implementation study.
- One representative of the involved Hospital personnel.
- One representative of the involved Primary care personnel.
- One IT or technology / eHealth expert.
- One health manager of the site.
- One patient.
- One project manager of the CCP protocol (who may be any of the above mentioned members or someone else).

Much attention has been given to the composition of these working teams, and additional members may be added to the teams to ensure at all times the required expertise for the appropriate development and deployment of the CCP protocol. However, being a member of a given working team does not preclude the participation in other working teams of the same site.

- Plan phase:

The first month of each PDSA cycle, will focus on the appropriate documentation of the planned interventions for the given implementation study, in the given site and cycle. To do so, a specific form has been created (**Annex I**). The form will be filled by each working team separately for each implementation study and site, capturing the specificities of each setting. The objectives of the cycle will always be in accordance to the CCP protocol spirit and guidelines. However, some specific objectives of the same implementation study may vary from one site to another. A description of the expected results from each intervention will be recorded, providing a background reference for the identification of unexpected results and/or unforeseen complications. The methodology for planned interventions will be defined and recorded in the form. Identification of any required additional participants (beyond working team members) will be based on the “small-scale testing” principle. Small groups of highly motivated volunteers can be selected in each site, preferably including both patients and professionals with an “early adopter” or “unafraid of change” profile. Paramount importance will be given to the description of how the working team will approach and recruit participants: detailed information on inclusion / exclusion criteria will be provided; appropriate information sheets to be given to each target group will be created; and, signed informed consent forms collected for all participants, according to the ethical and data protection specifications. A comprehensive list of the to-be-recorded variables will be established in line with the CCP protocol evaluation framework and its abovementioned dimensions. The “data over time” principle will need to be taken into account and repeated measures from previous PDSA cycles will be encouraged. Moreover, a core of common variables to be recorded in all sites in all PDSA cycles will be established in order to monitor the overall progress of the project and its integrated care deployment. All the selected variables will then be packed into a single database ready to be used. Finally, organizational aspects required for each given intervention will be specified, and detailed answers to the questions what; who; when; and how, will be provided. The project manager of each of the working teams will be the ultimate responsible for the fulfilment of the PDSA cycle documentation form.

Plan phase checklist:

□ Fulfilled PDSA cycle documentation form.

□ Creation of informed consent forms for participants.

□ Creation/adaptation of a database according to cycle’s requirements.

- Do phase:

The Do phase will begin after the completion of the PDSA cycle documentation forms and ideally be developed from M2 (i.e., Month 2) to M4 of each PDSA cycle. This phase will be ruled by the information on the documentation forms of each site. Organizational interventions, mock-tools or ready to be deployed technology will be tested. Data collection will be done using the databases created in the Plan phase. Great efforts will be exerted to ensure a comprehensive data collection, including the record of any unexpected issues or unplanned feedbacks. All collected information will be analyzed in the subsequent phase. The project manager of each of the working teams will be the ultimate responsible for the appropriate development of all activities of the Do phase as well as all data collection.

Do phase checklist:

□ Collection of signed informed forms.

□ Completed database.

- Study phase:

As soon as all the data from the Do phase will be available the Study phase will begin in order to analyze it and generate useful reports. This phase will be carried out around M5 of each PDSA cycle. The analyses in this phase will focus on monitoring the improvement and consolidating successful strategies through a predefined PDSA cycle evaluation form (**ANNEX II**), that considers the following evaluation categories: (i) Patients and professionals’ engagement and perspectives; (ii) New care models and supporting technology; (iii) Safety, ethical, and legal aspects; and, (iv) Maturity of the technology. Comparisons with predicted, baseline and/or previous results will be carried out as stated in the PDSA cycle documentation forms. The outcome of this phase will be a complete report on the results of the planed interventions in the given PDSA cycle, as well as the achieved progression towards CCP protocol goals. Unexpected issues and/or unplanned feedbacks from the Do phase will always be included in the reports.

Study phase checklist:

□ Study phase report, including:

□ Number and profile of participants in the PDSA cycle.

□ Detailed quantitative & qualitative results.

□ Unexpected issues or unplanned feedbacks.

- Act phase:

The Act phase will begin once the Study phase reports are available in each site. Study phase reports will be the base for decisions on adoption, adaptation or retraction of the studied interventions. Each site’s readiness for further implementation and scale-up of any given intervention will be discussed. All discussion in this phase will be held at two different levels: locally by each site’s working teams; and, globally at CCP protocol level. This phase will also entail the definition of future to-be-tested features or interventions, as well as the identification of any potential interactions that the implementation of an intervention could have in the site. The local and global consensus conclusions achieved in this phase will be the basis for the preparation of the next PDSA cycle’s Plan phase.

Act phase checklist:

□ Local discussion of Study phase results.

□ Global discussion of Study phase results.

□ Final consensus on adoption, adaptation or retraction of studied interventions.

□ Definition of future to-be-tested features or interventions.

# List of Abbreviations

CCP: Complex chronic patients

CHSS: Catalan Health Surveillance System

GMA: Adjusted Morbidity Groups

PDSA: Plan-Do-Study-Act

PIC: Patient Identification Number

# Supplementary references

1. Berwick, D. M., Nolan, T. W. & Whittington, J. The Triple Aim: Care, Health, And Cost. *Health Aff.* **27,** 759–769 (2008).

2. Whittington, J. W., Nolan, K., Lewis, N. & Torres, T. Pursuing the Triple Aim: The First 7 Years. *Milbank Q.* **93,** 263–300 (2015).

3. Vicky Serra-Sutton;, Espallargues, M. & Escarrabill, J. *Propuesta de indicadores para evaluar la atención a la cronicidad en el marco de la Estrategia para el Abordaje de la Cronicidad en el Sistema Nacional de Salud. Informe de evaluación de tecnologías sanitarias*. (2016).

4. Kelley, T. A. International Consortium for Health Outcomes Measurement (ICHOM). *Trials* **16,** O4 (2015).

5. Austin, P. C. An Introduction to Propensity Score Methods for Reducing the Effects of Confounding in Observational Studies. *Multivariate Behav. Res.* **46,** 399–424 (2011).

6. SELFIE. Sustainable intEgrated care modeLs for multi-morbidity: delivery, FInancing and performancE. Project n^o^ 634288 Available at: http://www.selfie2020.eu/.

7. Taylor, M. J., McNicholas, C., Nicolay, C., Darzi, A., Bell, D., *et al.* Systematic review of the application of the plan-do-study-act method to improve quality in healthcare. *BMJ Qual. Saf.* **23,** 290–8 (2014).

8. PADRIS. Programa públic d’analítica de dades per a la recerca i la innovació en salut. Available at: http://aquas.gencat.cat/ca/projectes/analitica_dades/.

# Figure Legends

Supplementary Figure 1

**Scheme of articulated datasets conforming the Catalan Health Surveillance System (CHSS).** Previously published in Dueñas-Espín I et al., BMJ Open. 2016 Apr 15;6(4) as part of the on-line supplementary material (Figure 1S) The articulation of the Table of clinical measurements (grey background) will progressively become operational through the PADRIS program^8^ launched in January 2017. GMA: Adjusted Morbidity Groups, PIC: Personal identification codes.

# ANNEX I

## PDSA cycle documentation form

| **Implementation study** | |  | | | **Site** |  | | |
| --- | --- | --- | --- | --- | --- | --- | --- | --- |
| **Cycle** | |  | **Start date** |  | | | **End date** |  |
| **Objectives** |  | | | | | | | |
| **Expected results** |  | | | | | | | |
| **Methods** | Participants: | | | | | | | |
|  | Data to be collected: | | | | | | | |
|  | Organizational aspects: | | | | | | | |

# ANNEX II

## PDSA cycle Evaluation form

| **Implementation study** |  | | | **Site** |  | | |
| --- | --- | --- | --- | --- | --- | --- | --- |
| **Cycle** |  | **Start date** |  | | | **End date** |  |
| **Patients and professionals’ engagement and perspectives** | | | | | | | |
| - 1. All the professionals participating in the site study management were involved:   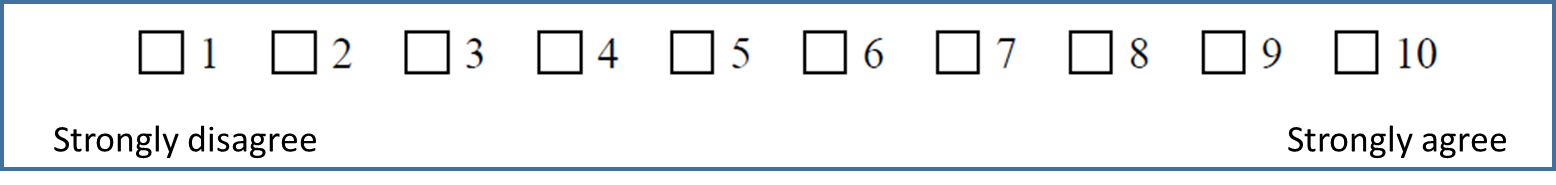   - 1. Your contributions have been taken into account in the design process:   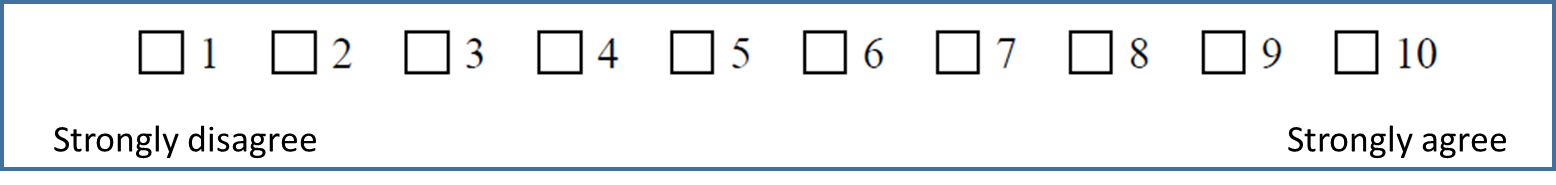   - 1. The working methodology so far has been appropriate:   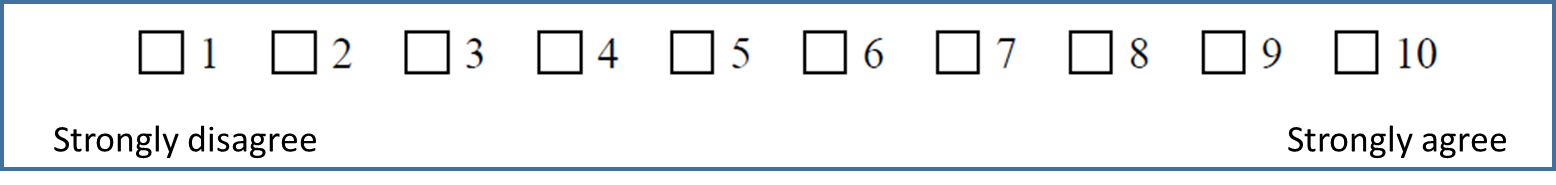   - 1. At this point, the site study would fulfill the professionals’ expectations:   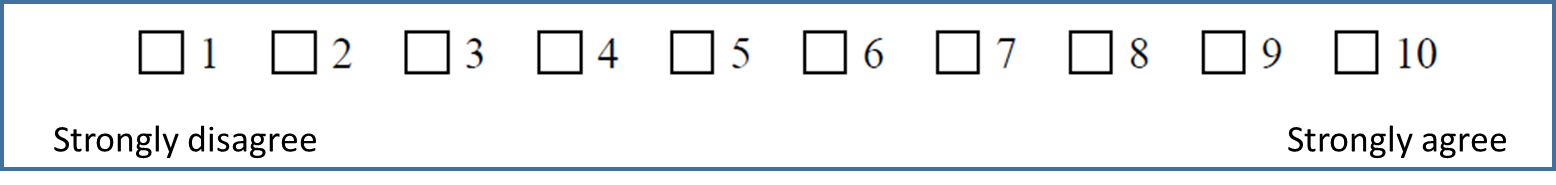   - 1. At this point, the site study would fulfill the patients’ expectations:   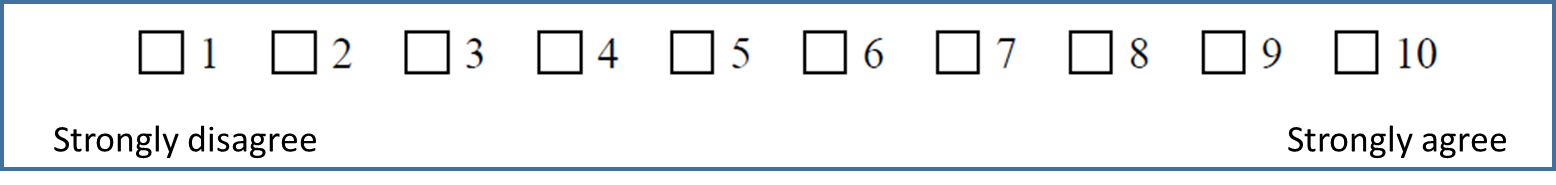 | | | | | | | |
| **New care models and supporting technology** | | | | | | | |
| - 1. The site study workflow is well-defined:   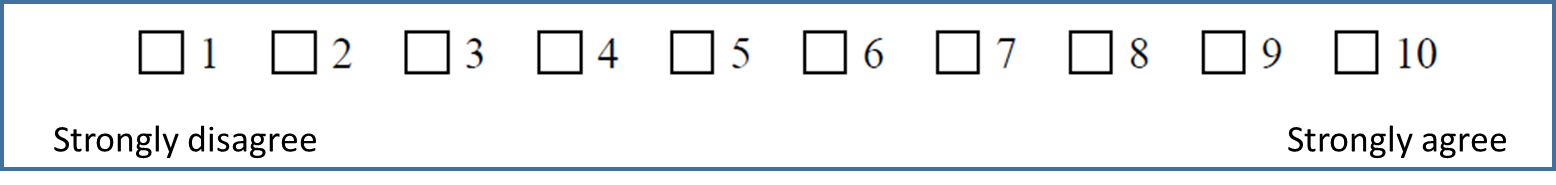   - 1. The proposed stratification and risk assessment tools could improve daily clinical practice:   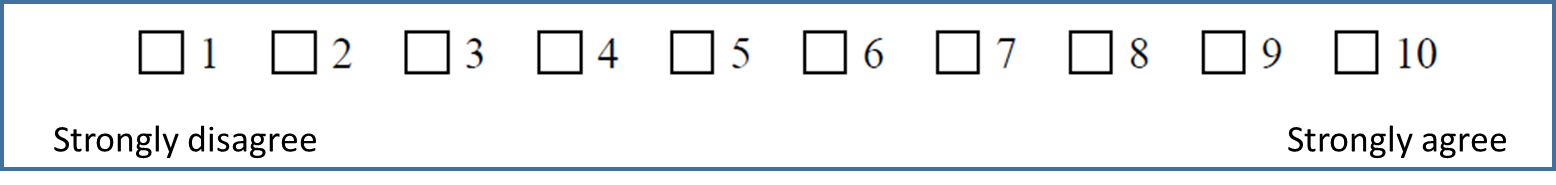   - 1. The deployment of the new care model could improve daily clinical practice:   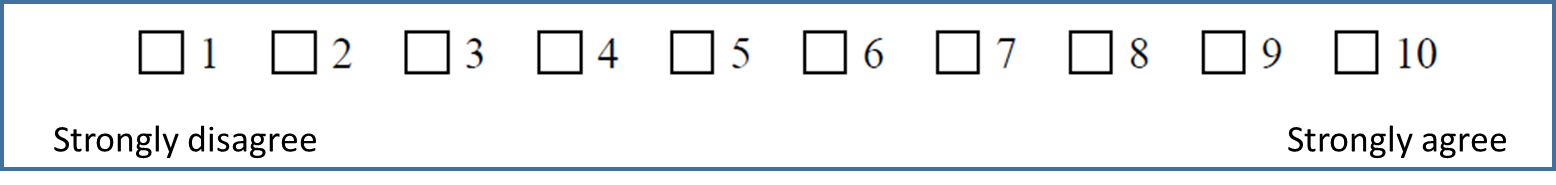   - 1. The proposed technological solutions could improve daily clinical practice:   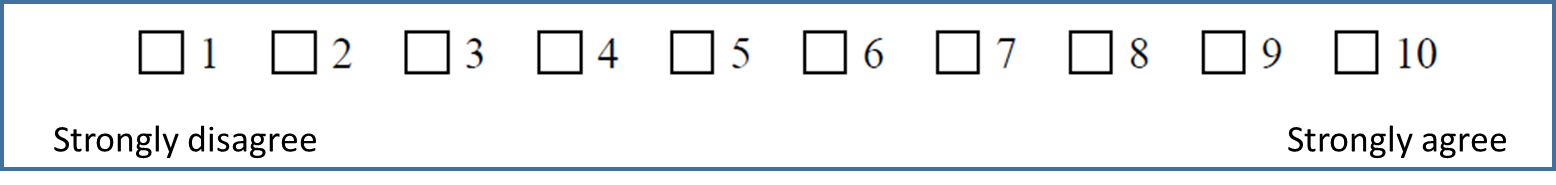   - 1. The proposed technological solutions would cover all your expected needs:   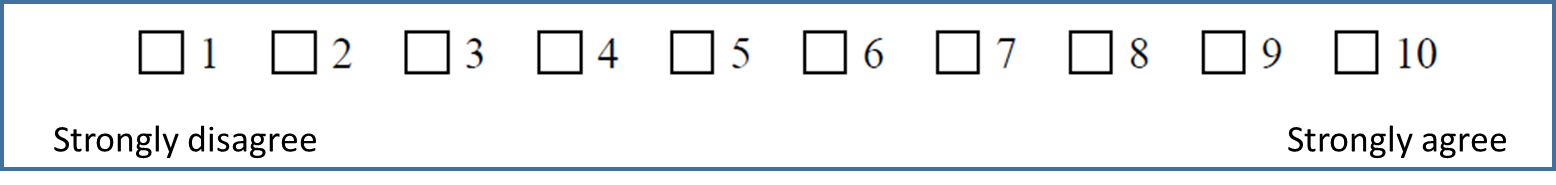 | | | | | | | |
| **Safety, ethical, and legal aspects** | | | | | | | |
| - 1. The new care model would not endanger the professionals or patients:   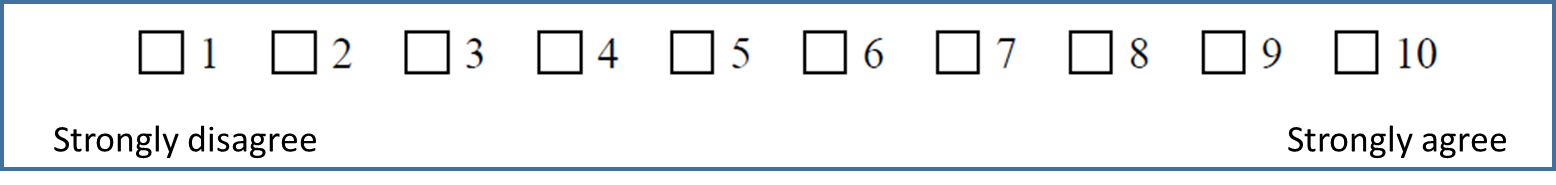   - 1. You do not perceive threats concerning how the information in the supporting technological systems will be handled:   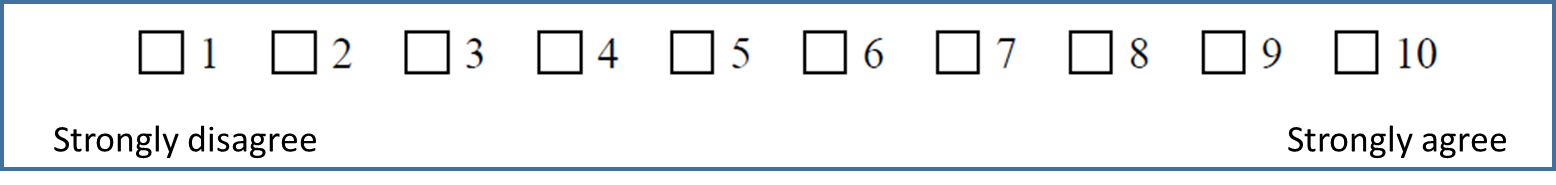 | | | | | | | |
| **Maturity of the technology** | | | | | | | |
| - 1. The new care model is ready to be deployed at your working site:   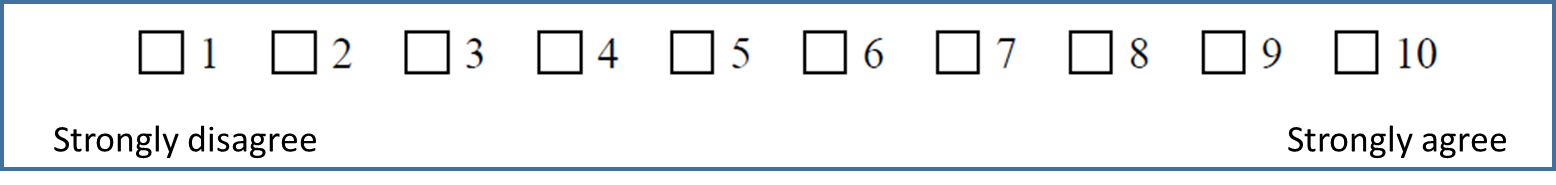   - 1. The proposed workflow is ready to be deployed at your working site:   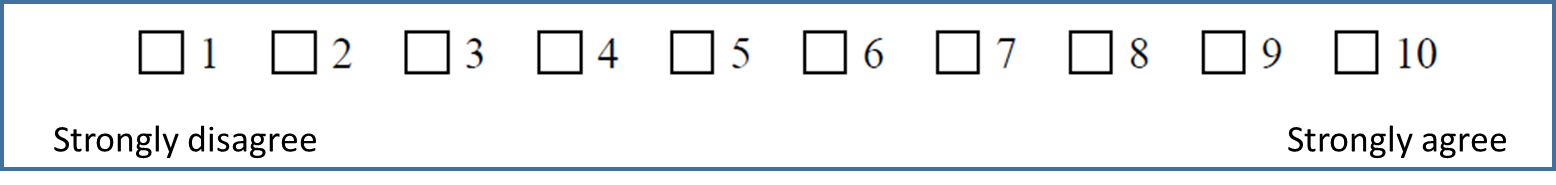   - 1. The proposed technological support is ready to be used at your working site:   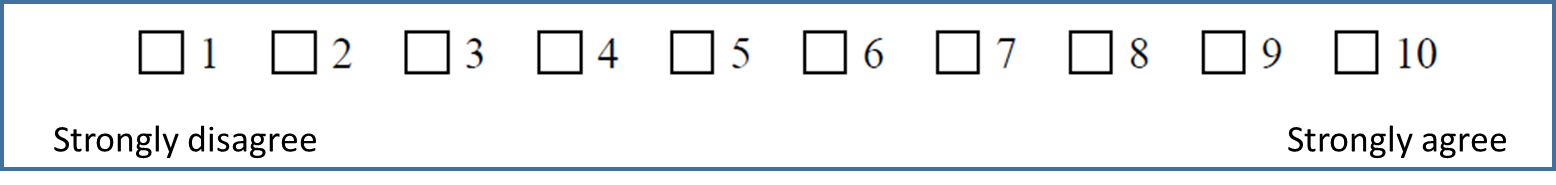   - 1. The new care model is ready to be deployed in other than the CCP protocol sites:   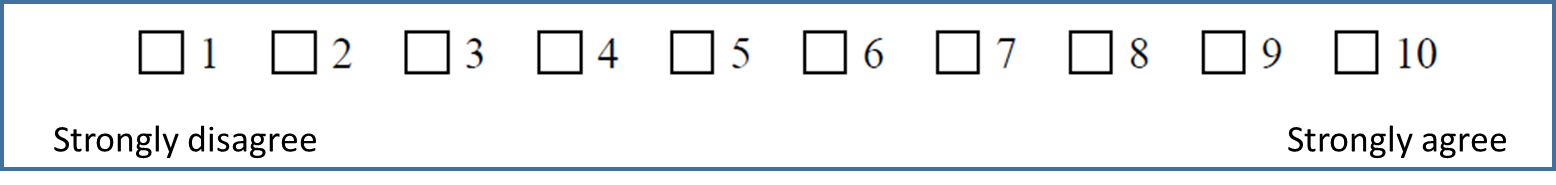 | | | | | | | |
