# Supplementary figures and images for: Protocol for regional implementation of community-based collaborative management of complex chronic patients
Source: NPJ Prim Care Respir Med. 2017 Jul 14;27:44. doi: 10.1038/s41533-017-0043-9 (PMC5511202; doi:10.1038/s41533-017-0043-9)

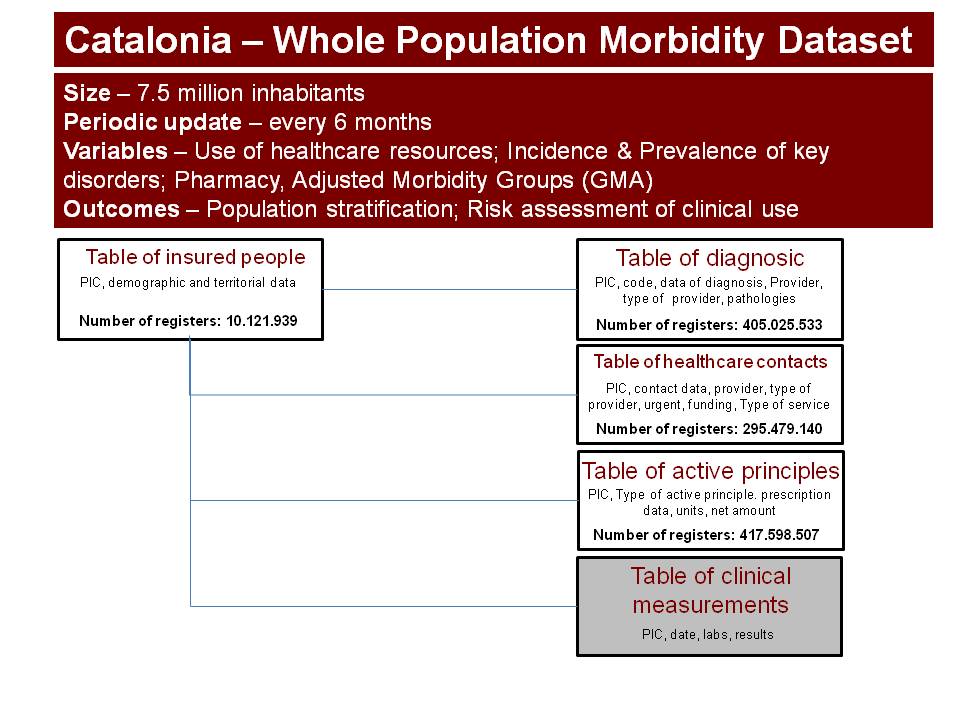

Supplement: Supplementary file 2 — Supplemetary Figure 1 [file 41533_2017_43_MOESM2_ESM.docx]
